# Control Control Control: A Reassessment and Comparison of GenBank and Chromatogram mtDNA Sequence Variation in Baltic Grey Seals (*Halichoerus grypus*)

**DOI:** 10.1371/journal.pone.0072853

**Published:** 2013-08-16

**Authors:** Katharina Fietz, Jeff A. Graves, Morten Tange Olsen

**Affiliations:** 1 Centre for GeoGenetics, Natural History Museum of Denmark, Copenhagen, Denmark; 2 School of Biology, University of St Andrews, St Andrews, Scotland, United Kingdom; 3 Department of Bioscience, Aarhus University, Roskilde, Denmark; University of Canterbury, New Zealand

## Abstract

Genetic data can provide a powerful tool for those interested in the biology, management and conservation of wildlife, but also lead to erroneous conclusions if appropriate controls are not taken at all steps of the analytical process. This particularly applies to data deposited in public repositories such as GenBank, whose utility relies heavily on the assumption of high data quality. Here we report on an in-depth reassessment and comparison of GenBank and chromatogram mtDNA sequence data generated in a previous study of Baltic grey seals. By re-editing the original chromatogram data we found that approximately 40% of the grey seal mtDNA haplotype sequences posted in GenBank contained errors. The re-analysis of the edited chromatogram data yielded overall similar results and conclusions as the original study. However, a significantly different outcome was observed when using the uncorrected dataset based on the GenBank haplotypes. We therefore suggest disregarding the existing GenBank data and instead using the correct haplotypes reported here. Our study serves as an illustrative example reiterating the importance of quality control through every step of a research project, from data generation to interpretation and submission to an online repository. Errors conducted in any step may lead to biased results and conclusions, and could impact management decisions.

## Introduction

Genetic data provides a powerful tool for the study of living organisms and finds increasing use within the disciplines of evolution, ecology, population biology, conservation, and management [1]. Over the years, the use and development of genetic approaches have resulted in the generation of large amounts of genetic data, which has been made publically available in repositories such as GenBank [2], providing a unique and very valuable resource for the research community. The utility of such public data, produced by others and from several different researchers, relies heavily on the assumption of high data quality [3]. However, although much has been accomplished in terms of minimizing their prevalence, sequence errors are still an important issue for both Sanger and next generation sequencing data [4–7].

In our ongoing study of grey seal population dynamics we were interested in using the information of Graves et al. [8] and the corresponding mtDNA haplotype data in GenBank to recreate their mtDNA dataset. A closer examination of the GenBank data revealed that several of the haplotypes in the GenBank repository were identical. To examine these inconsistencies and to uncover other potential issues, the original chromatogram files generated by Graves and co-authors were re-edited and re-analysed independently. Here we report on the steps performed as part of this reassessment, provide information on the re-edited data, and discuss the implications of our findings.

## Materials and Methods

### Datasets

The reassessment was based on three different datasets: i) the 40 grey seal haplotypes posted in GenBank (accession numbers AM287215-AM287254) by Graves et al. [8]; ii) the raw ABI chromatograms from Graves et al. [8], covering three different grey seal breeding sites in the Baltic Sea: the Bay of Bothnia (BB), Estonia (EST), and the Stockholm Archipelago (STA); and iii) an “erroneous” dataset constructed from the GenBank haplotypes and the information on haplotype distribution in Table 5 of the Graves et al. study [8]. Specifically, we first downloaded the haplotypes listed in GenBank and assembled them with zero mismatches in order to assess the actual number and types of haplotypes in the data listed in GenBank. Second, these haplotypes were checked against the re-edited dataset which was obtained by manually checking all raw chromatograms, changing errors in base calls and omitting poor quality chromatograms (i.e. those in which one third or more of the nucleotides could not be scored consistently). Re-editing of the chromatograms was performed by two people independently and all initial data processing was performed in Geneious 6.0.4 [9]. Third, we constructed an “erroneous” dataset based on the GenBank haplotypes and their distribution as reported in Table 5 of Graves et al. [8], where haplotypes 1 through 40 in GenBank were assumed to correspond to haplotypes 1 through 40 in Table 5. This latter dataset was constructed in order to assess the potential implications of not correcting the GenBank data.

### Data analysis

In order to assess whether the conclusions of the previously published results are still valid, we reanalysed the erroneous data and the re-edited data, respectively, using the same approach as in the Graves et al. study [8]. Specifically, the number of unique control region haplotypes, haplotype frequencies and distribution, number of polymorphic sites, nucleotide composition, haplotype diversity, and nucleotide diversity were estimated using Arlequin 3.5 [10]. A Chi^2^ test using SPSS v. 19 [11] was used to check for possible differences between the three breeding sites in the proportion of haplotypes unique to each site. Analysis of Molecular Variance (AMOVA) using Arlequin 3.5 was used to re-examine overall and pairwise spatial heterogeneity among breeding sites. Further, since the microsatellite data in the original study suggested significant genetic differentiation between the seals in STA and the two other breeding sites [8], we pooled the BB and EST samples and performed AMOVAs for this and the two other combinations (i.e. BB-STA and EST-STA). Finally, the results published in the original study and the results of the erroneous dataset were tested against the results based on the re-edited dataset. Comparisons were made for the haplotype and nucleotide diversities, as well as the proportion of unique haplotypes per breeding area, using 95% confidence intervals (CI) and Chi^2^ tests, respectively. Moreover, to illustrate potential differences in the distribution of haplotypes, we constructed haplotype networks for the re-edited and the erroneous datasets using the program TempNet [12].

## Results

### Quality control of GenBank haplotypes

**Figure 1:**
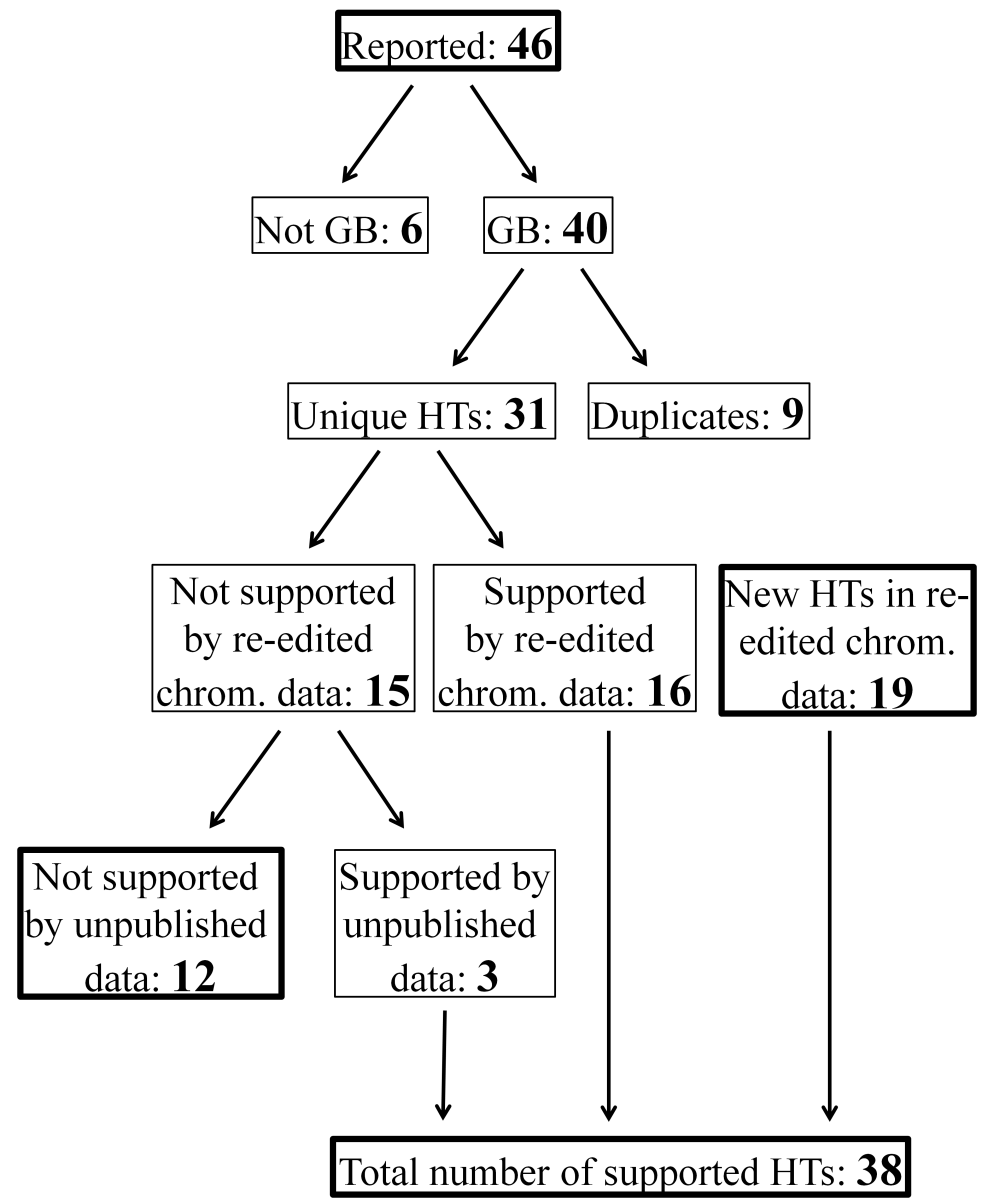
Workflow for quality control of the original GenBank haplotypes and the re-edited chromatograms. HT = Haplotype; GB = GenBank.

In the Graves et al. study a total of 46 different haplotypes were reported (Table 5 in [8]). However, only 40 haplotypes were posted in GenBank and assembly of these 40 sequence files revealed nine pairs of identical sequences (i.e. duplicates) and only 31 different haplotypes (Table 1, Table 2, Figure 1). Of these, 16 were supported by the re-edited chromatograms, while 15 of the haplotypes in GenBank were not supported. Further examination of these unsupported 15 haplotypes revealed three matches against an unpublished grey seal dataset from Denmark (Fietz et al., unpublished), implying that 20% (3/15) of the unsupported haplotypes could turn out to be false negatives. Overall, the total number of haplotypes posted in GenBank that could be supported by chromatogram files was 19 (16 + 3). This corresponds to 61.3% of the 31 different haplotypes listed in GenBank. In addition however, 19 new haplotypes were discovered in the re-edited chromatograms in addition to those already listed in GenBank, resulting in a total number of 38 grey seal haplotypes (Table 2, Figure 1).

**Table 1:** Overview of haplotype duplicates in GenBank.

**Duplicate**	**GenBank Haplotype ID**	**GenBank Accession Number**
1	31, 32, 37, 40	AM287245, AM287246, AM287251, AM287254
2	19, 21, 29	AM287233, AM287235, AM287243
3	5, 28	AM287219, AM287242
4	16, 25	AM287230, AM287239
5	11, 17	AM287225, AM287231
6	7, 9	AM287221, AM287223

**Table 2:** The distribution of grey seal haplotypes based on the re-edited chromatograms.

**New HT ID**	**BB**	**EST**	**STA**	**Baltic Total**	**Unpublished data**	**Old HT ID**
**1**	4	5	3	12	14	5, 28
**2**	4	4	3	11	2	19, 21, 29
**3**	3	3	3	9	3	New
**4**	1	3	5	9	5	23
**5**	3	3		6	3	27
**6**	2	1	2	5	16	38
**7**	3	1		4	3	New
**8**	1	1	1	3		New
**9**	2	1		3		14
**10**	1	1	1	3		New
**11**	1	1	1	3	2	New
**12**	2	1		3		11, 17
**13**	1	2		3	2	16, 25
**14**			2	2	1	New
**15**		2		2		New
**16**	1	1		2		24
**17**			2	2	2	New
**18**	1	1		2	4	7, 9
**19**	1	1		2		18
**20**			2	2		New
**21**	1			1		12
**22**	1			1		15
**23**	1			1		20
**24**	1			1	1	New
**25**	1			1	1	New
**26**	1			1	1	New
**27**	1			1		New
**28**	1			1	1	New
**29**		1		1		22
**30**		1		1		New
**31**		1		1		New
**32**		1		1		New
**33**			1	1		New
**34**			1	1	1	New
**35**			1	1		3
**36**					3	31, 32, 37, 40
**37**					20	34
**38**					4	35
**Total**	**39**	**36**	**28**	**103**		

Haplotypes 36-38 were identical to haplotypes originally posted in GenBank and supported by our unpublished data, but not by the re-edited chromatograms.

Old HT ID corresponds to the original haplotypes posted in GenBank by Graves et al. [8].

HT = Haplotype; BB = Bay of Bothnia; EST = Estonia; STA = Stockholm Archipelago.

### Analysis and comparison of datasets

The length of the mtDNA fragment in the re-edited chromatogram dataset was reduced from 489 bp to 435 bp and the number of grey seal samples reduced from 114 to 103 grey seals (Table 2). The nucleotide composition was 26.8% cytosine, 28.6% thymine, 26.2% adenine, and 18.4% guanine (45.2% GC content). A total of 37 polymorphisms were identified, resulting in 35 unique haplotypes (Table 2) and an overall nucleotide diversity (π) of 0.017 ± 0.001 SD. The number of haplotypes, π, haplotype diversity, and the 95% confidence intervals (CI) for each breeding site are listed in Table 3. The two most common haplotypes are found in 11.6% and 10.6% of the seals analyzed, respectively (Table 2). Eight haplotypes were found in all three breeding sites, a further eight were found in two of the breeding sites, and 19 (54.3%) were unique to one site. The proportion of haplotypes in a specific site that were unique was 33.3% for BB, 23.8% for EST and 42.9% for STA, respectively, and did not differ significantly among the three sites (χ^2^ = 1.42, *P* = 0.490). The AMOVA suggested an absence of genetic differentiation among breeding sites both overall (*F*_ST_ = 0.000; *P* = 0.822) and in the pairwise tests (Table 4). Low but non-significant genetic differentiation was detected between STA and the pooled BB-EST samples (*F*_ST_ = 0.016, *P* = 0.344), whereas there was an absence of genetic variation when pooling EST-STA (*F*_ST_ = 0.000; *P* = 0.660) and BB-STA (*F*_ST_ = 0.000, *P* = 1.000).

**Table 3:** Number of haplotypes, haplotype diversity, nucleotide diversity (π), and 95% confidence intervals (CI) for each sample site estimated for the original, the re-edited and the erroneous datasets.

	**Population**	**N**	**Number of haplotypes**	**Haplotype diversity (95% CI)**	**Π (95% CI)**
**Original dataset**	**Bay of Bothnia**	40	18	0.968 (0.941, 0.995)	0.015 (0.000, 0.043)
	**Estonia**	40	26	0.965 (0.940, 0.990)	0.016 (0.000, 0.034)
	**Stockholm**	34	23	0.943 (0.904, 0.982)	0.015 (0.000, 0.031)
**Re-edited dataset**	**Bay of Bothnia**	39	24	0.968 (0.942, 0.993)	0.018 (0.000, 0.036)
	**Estonia**	36	21	0.957 (0.925, 0.990)	0.018 (0.000, 0.036)
	**Stockholm**	28	14	0.939 (0.894, 0.984)	0.016 (0.000, 0.033)
**Erroneous dataset**	**Bay of Bothnia**	40	21	0.958 (0.931, 0.985)	0.017 (0.015, 0.019)
	**Estonia**	40	21	0.958 (0.931, 0.985)	0.060 (0.058, 0.062)
	**Stockholm**	28	11	0.910 (0.859, 0.961)	0.017 (0.015, 0.018)

Degrees of freedom = 2.

**Table 4:** Genetic differentiation among Baltic grey seal breeding sites estimated for the re-edited and the erroneous datasets.

		**Bay of Bothnia**	**Estonia**	**Stockholm**
**Re-edited dataset**	**Bay of Bothnia**	*	0.997	0.245
	**Estonia**	0.000	*	0.602
	**Stockholm**	0.006	0.000	*
**Erroneous dataset**	**Bay of Bothnia**	*	0.807	0.294
	**Estonia**	0.000	*	0.558
	**Stockholm**	0.004	0.000	*

Pairwise *F*_ST_ values for the three sample sites are below the diagonal and *P*-values are above. Pairwise *F*_ST_ values were estimated but not reported in Graves et al. [8] and hence not included in this table.

The same analyses were conducted with the erroneous dataset consisting of 108 grey seals: 40 individuals from BB, 40 individuals from EST, and 28 individuals from STA. The nucleotide composition was 28.3% cytosine, 28.3% thymine, 26.2% adenine, and 17.1% guanine (45.5% GC content). A total of 39 polymorphic sites were identified, resulting in 31 unique haplotypes and an overall nucleotide diversity (π) of 0.017 ± 0.001 SD. The number of haplotypes, π, haplotype diversity, and the 95% confidence intervals (CI) for each breeding site are listed in Table 3. The two most common haplotypes are found in 14.0% and 11.9% of the seals analyzed, respectively. Seven haplotypes were found in all three breeding sites, a further seven were found in two of the breeding sites, and 26 (65.0%) were unique to one site. The proportion of haplotypes in a specific site that were unique was 50.0% for BB, 47.8% for EST and 16.7% for STA, respectively, and did not differ significantly among the three sites (χ^2^ = 4.14, *P* = 0.126). The AMOVA suggested an absence of genetic differentiation among breeding sites both overall (*F*_ST_ = 0.000; *P* = 0.586) and in the pairwise tests (Table 4). No genetic differentiation was detected when pooling BB-EST (*F*_ST_ = 0.000; *P* = 0.606), EST-STA (*F*_ST_ = 0.000; *P* = 0.609) and BB-STA (*F*_ST_ = 0.000, *P* = 0.593).

**Figure 2:**
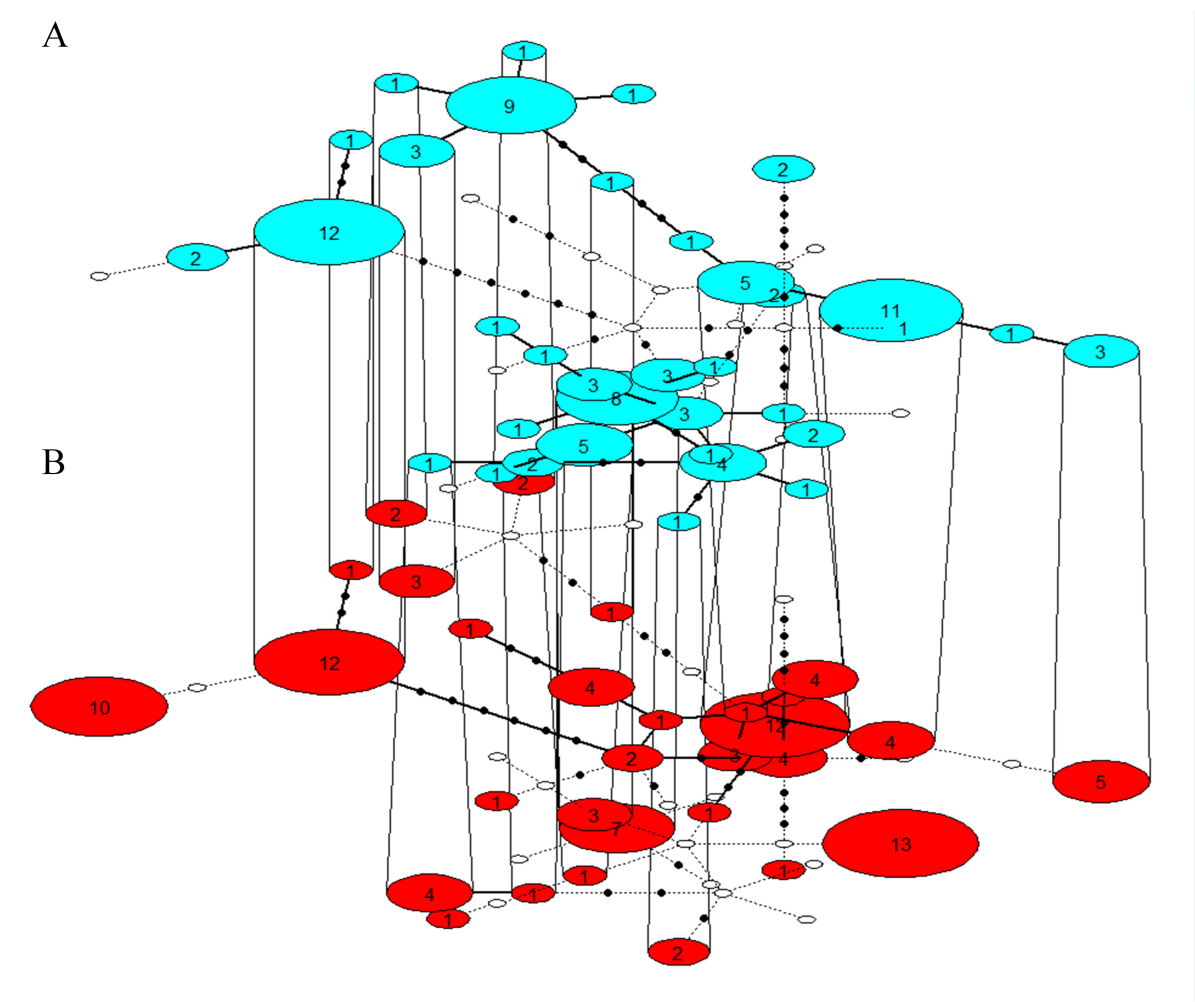
Haplotype networks displaying the distribution of haplotypes in the re-edited (A; blue) and in the erroneous (B; red) dataset. Size and number in coloured circles represent occurrence of haplotypes. Shared haplotypes in both networks are connected by lines. A black dot symbolizes a haplotype not observed in either dataset, but indicates a parsimonious path between haplotypes. A white dot illustrates a haplotype present in one network, but missing in the other.

The two haplotype networks differed markedly in the distribution and occurrence of haplotypes with several of the most frequent haplotypes in one dataset missing in the other dataset (Figure 2). Despite this, our comparison of the published results and the results generated by re-editing and analysing the data did not suggest significant differences. That is, the published and the re-estimated haplotype and nucleotide diversities, as well as the proportion of haplotypes unique to a single breeding site (χ^2^ =5.70, *P* = 0.058), were statistically similar. In the erroneous dataset however, the nucleotide diversity in EST was significantly higher than in the re-edited dataset (two-tailed t-test; *t* = 4.467; *P*=0.047), and the proportion of haplotypes unique to a single breeding site also differed significantly from the re-edited dataset (χ^2^ =13.20, *P* = 0.001).

## Discussion

The main issue detected by our reassessment of the mtDNA data generated by Graves et al. [8] relates to the number and type of haplotypes listed in GenBank, and to a minor degree, the editing and scoring of raw chromatogram files (Table 5). In the present case, the mistake was readily detected since only 40 of 46 reported haplotypes were posted in GenBank and nine of these proved to be duplicates (Figure 1). Our re-analyses showed that the biological significance of these mistakes was minor, thus the conclusions drawn by Graves et al. regarding mtDNA genetic diversity and differentiation within the Baltic are still valid [that levels of genetic differentiation among the three Baltic breeding sites are low, but slightly higher between STA and the two other breeding sites (BB and EST), as also suggested by the microsatellite data in Graves et al. [8]]. However, our assessment also revealed that, had someone reconstructed a dataset based on the GenBank data and used this in combination with their own data, they would have obtained biased estimates of the magnitude and distribution of genetic diversity. Such bias is likely to have had severe implications for estimates of divergence time, effective population size and migration rates. This reiterates the importance of quality control through all steps of a project; from generating the data to making it publicly available in e.g. GenBank [3–7]. Errors in any of those steps may lead to wrong results and conclusions, which in turn could lead to biased management and conservation decisions with negative consequences for the population and/or species of concern. In order to minimize such potential effects we urge researchers to conduct appropriate controls of their own and others data. Indeed, such quality control is paramount for the usefulness of data repositories such as GenBank. With regards to the grey seal mtDNA data, we suggest that future studies should disregard the existing GenBank files (accession numbers AM287215-AM287254) and instead using the 38 haplotypes found by re-editing of the Graves et al. chromatogram files, many of which were also confirmed by a yet unpublished dataset from Denmark. These 38 new haplotypes may serve as a valuable reference for future genetic studies of grey seals (accession numbers KF483184-KF483221).

**Table 5:** Main findings of the analyses of the respective datasets.

GenBank Haplotypes	- Only 40 haplotypes listed in GenBank (46 reported in Graves et al.)
	-Nine of the haplotypes were duplicates
	-Sixteen of the haplotypes were supported by the re-edited dataset
Re-edited dataset compared to the original dataset	- Fewer haplotypes and slightly smaller dataset
	- Slightly higher difference between STA and the other breeding sites
	- Lower proportion of unique haplotypes in BB and EST
Erroneous dataset compared to the re-edited and original Datasets	- Very different type and frequency of mtDNA haplotypes
	- Higher nucleotide diversity in EST
	- Slightly lower haplotype diversity in STA
	- Much lower proportion of unique haplotypes in STA
